# Implementing tobacco use treatment guidelines in community health centers in Vietnam

**DOI:** 10.1186/s13012-015-0328-8

**Published:** 2015-10-09

**Authors:** Donna Shelley, Nancy VanDevanter, Charles C. Cleland, Linh Nguyen, Nam Nguyen

**Affiliations:** Department of Population Health, New York University School of Medicine, 227 East 30th Street, 7th floor, New York, NY 10016 USA; New York University College of Nursing, 433 First Ave, New York, NY 10010 USA; Center for Drug Use and HIV Research, New York University College of Nursing, 433 First Ave, New York, NY 10010 USA; Institute of Social Medical Studies, No. 18, Lot 12B, Trung Yen 10, Trung Hoa, Cau Giay District, Hanoi, Vietnam

**Keywords:** Vietnam, Clinical practice guidelines, Treatment of tobacco dependence, Tobacco cessation

## Abstract

**Background:**

Vietnam has a smoking prevalence that is the second highest among Southeast Asian countries (SEACs). According to the World Health Organization (WHO), most reductions in mortality from tobacco use in the near future will be achieved through helping current users quit. Yet, largely due to a lack of research on strategies for implementing WHO-endorsed treatment guidelines in primary care settings, services to treat tobacco dependence are not readily available to smokers in low middle-income countries (LMICs) like Vietnam. The objective of this study is to conduct a cluster randomized controlled trial that compares the effectiveness of two system-level strategies for implementing evidence-based guidelines for the treatment of tobacco use in 26 public community health centers (CHCs) in Vietnam.

**Methods/Design:**

The current study will use a cluster-randomized design and multiple data sources (patient exit interviews, provider and village health worker (VHW) surveys, and semi-structured provider/VHW interviews) to study the process of adapting and implementing clinical practice guidelines in Vietnam and theory-driven mechanisms hypothesized to explain the comparative effectiveness of the two strategies for implementation. CHCs will be randomly assigned to either of the following: (1) training plus clinical reminder system (TC) or (2) TC + referral to a VHW (TCR) for three in person counseling sessions. The primary outcome is provider adherence to tobacco use treatment guidelines. The secondary outcome is 6-month biochemically verified smoking abstinence.

**Discussion:**

The proposed implementation strategies draw on evidence-based approaches and a growing literature that supports the effectiveness of integrating community health workers as members of the health care team to improve access to preventive services. We hypothesize that the value of these implementation strategies is additive and that incorporating a referral resource that allows providers to delegate the task of offering counseling (TCR) will be superior to TC alone in improving delivery of cessation assistance to smokers. The findings of this research have potential to guide large-scale adoption of promising strategies for implementing and disseminating tobacco use treatment guidelines throughout the public health system in Vietnam and will serve as a model for similar action in other LMICs.

**Trial registration:**

NCT01967654

**Electronic supplementary material:**

The online version of this article (doi:10.1186/s13012-015-0328-8) contains supplementary material, which is available to authorized users.

## Background

Almost half of adult men in Vietnam are current smokers, a smoking prevalence that is the second highest among Southeast Asian countries (SEACs) [1]. If current smoking rates are not addressed, it is estimated that in 10 years, tobacco use will be responsible for about 25 % of adult male deaths in Vietnam [2]. Most reductions in mortality from tobacco use in the near future will be achieved through helping current users quit [3, 4]. Encouragingly, two thirds of current smokers in Vietnam are planning to or thinking about quitting and over half attempt to quit annually [1].

**Fig. 1:**
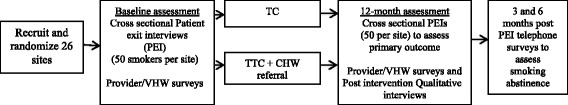
Study design

**Fig. 2:**
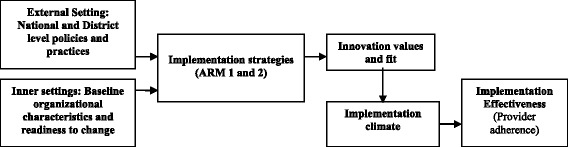
Conceptual framework

Tobacco use treatment, as defined by the U.S. Preventive Health Service Guideline (PHS Guideline) on *Treating Tobacco use and Dependence*, is evidence-based and highly cost-effective [5]. The Guideline, which is endorsed by the World Health Organization (WHO), is based on a meta-analysis of over 8000 studies and provides strong evidence that asking all patients about tobacco use, advising smokers to quit, assessing readiness, providing assistance, and arranging follow-up (the 5As) can significantly increase smoking abstinence rates [5]. Yet, adoption of guideline-recommended care into routine public health and clinical practice in low middle-income countries (LMICs) is suboptimal [6, 7]. This is in large part due to a lack of research on strategies for implementing evidence-based tobacco use treatment guidelines.

Implementing evidence-based tobacco use treatment is a core provision in the WHO Framework Convention on Tobacco Control (FCTC). The FCTC is an evidence-based treaty that was developed by the WHO in response to the globalization of the tobacco epidemic [8]. The FCTC regulatory strategies include implementing evidence-based smoking cessation treatment in public health care delivery settings. In fact, Article 14 of the FCTC states that “each country shall take effective measures to promote cessation and adequate treatment for tobacco dependence” [9]. Although Vietnam has a strong public health delivery system, according to the 2010 Global Adult Tobacco Survey, like other SEACs, services to treat tobacco dependence are not readily available to smokers [1]. Barriers to integrating treatment into routine primary care in LMICs are similar to those in the USA and include the following: (1) inadequate training of health care providers, (2) lack of evidence-based systems for implementing guideline recommended care, and (3) a lack of research on strategies for implementing tobacco use treatment guidelines in LMICs [10].

Closing the gap between research and practice is stymied by limited research on cost-effective strategies for integrating cessation services into routine practice. Drawing upon a burgeoning implementation science literature [11–17] and a growing literature that supports the effectiveness of integrating community health workers as members of the health care team to improve access to preventive services [18–24], we propose to compare the effects of two organization-level strategies: (1) training and a clinical reminder system (TC) vs. (2) TC + referral (TCR) to community health workers (CHW), referred to as village health workers (VHW) in Vietnam, for additional counseling and support. The PHS Guideline strongly recommends staff training and clinical reminder systems as the foundation for increasing adherence to guideline-recommended care [5]. However, several studies have shown that adding a referral system can enhance rates of provider adherence to tobacco use treatment guidelines and increase smoking abstinence rates beyond that of training and clinical reminders alone [13–16]. The impact of referral systems on adherence to tobacco use treatment guidelines is also supported by a recent study in Malaysia [25]. This study, conducted in diabetes clinics, similarly found that providing a referral to stand-alone cessation clinics motivated clinicians to routinely provide cessation advice. Therefore, offering a referral option for additional counseling may enhance quit attempts and cessation rates. However, the Malaysian model of creating stand-alone cessation clinics is too expensive to disseminate widely and stands in contrast to recommendations from the WHO’s recently published guidelines for implementing Article 14 which states: “In order to promote tobacco cessation and develop tobacco dependence treatment as rapidly as possible and at as low a cost as possible, countries should use existing resources and infrastructure” [9].

This proposal leverages existing infrastructure elements, including a robust public health care delivery system with an extensive network of VHWs in Vietnam. In Vietnam, as in other LMICs, VHWs have a strong track record of effectively delivering preventive services and increasing the reach of these programs [18, 20]. As a critically important member of the public health care system, it is surprising that in LMICs, there are no studies evaluating the role of community health workers as a referral resource for increasing access to evidence-based smoking cessation services, and we are aware of only one study in the USA [19]. Consistent with WHO Guidelines for implementing Article 14, CHWs offer a sustainable resource for ensuring wide access to support for tobacco users who wish to quit. Using a two-arm cluster randomized control trial design, the aims of this study are to (1) compare the effectiveness and cost-effectiveness of two multi-component strategies for implementing tobacco use treatment guidelines and (2) use a mixed methods approach to explore potential theory-driven mechanisms hypothesized to explain the comparative effectiveness of the implementation strategies.

## Methods

### Study setting

The Vietnamese health care system is hierarchically organized into four administrative levels: central, province, district, and community. At the central level is the Ministry of Health (MOH), the main national authority in the health sector, which formulates and implements national health policies and programs. The provincial-level health system consists of Provincial Health Departments and Preventive Health Centers, which are administered by the Provincial People’s Committee in each province. At the district level, the District People’s Committee administers district health centers and district-level hospitals. Within districts, the community health centers (study sites for this research) serve as the primary access point for public health and preventive care services in Vietnam, each providing services for an average of 3000–15,000 people in their surrounding community. CHCs are charged with implementing more than 10 national health programs, treatment of common diseases, provision of health counseling and education, referral services, pre- and post-natal care, family planning, and food hygiene and safety. Each CHC is staffed by 5–6 clinicians, including 1 physician and 3–5 other health professionals (nurses, midwives). In addition, each CHC is supported by a network of 8–20 VHWs who provide counseling and education to implement national health programs at the village level and serve as clinician extenders to ensure patients are adhering to clinician-recommended care. VHWs are under the direct management and direction of the CHCs and coordinate with community and social organizations in the village.

### Study design

We are conducting a two-arm, cluster randomized controlled trial comparing (1) training and clinical reminder system (TC) vs. (2) TC + referral to a VHW (TCR) for additional counseling (Fig. 1). The primary outcome is improvement in provider adherence to tobacco use treatment guidelines which has been found through extensive meta-analysis to be an essential determinant of patient cessation outcomes [5]. The secondary outcome is 6-month biochemically verified smoking abstinence rates. Finally, guided by Damschroeder’s Consolidated Framework for Implementation Research and Weiner’s organizational model of innovation implementation, we will explore external (e.g., national and district level policy) and internal setting constructs (e.g., organizational readiness) that may influence the relationship between the implementation strategies and implementation effectiveness [26–29].

### Study site eligibility and recruitment

We are recruiting 26 health centers in Thai Nguyen, a rural province north of Hanoi. Thai Nguyen has 9 districts with 180 CHCs. Site criteria include having at least one physician, ≥4 allied health care professional staff, ≥5 VHWs, and a patient population of at least 3000. Site recruitment started with in-person site visits with the Director of the District Health Centers to introduce the study and to obtain a list of CHCs that fit these criteria. Among those expressing interest, we randomly selected 26 CHCs. For practical reasons (cost and staffing), clinic sites will be recruited in 3 successive waves with 8 in the first wave, 10 in the second, and 8 in the third wave.

### Conceptual framework

Our proposed study draws on the Consolidated Framework for Implementation Research (CFIR) to inform the analysis of factors that influence effective implementation (i.e., provider adherence to tobacco use treatment guidelines) and Weiner’s model for implementation effectiveness [26, 27, 29] (Fig. 2). Similar to the socio-ecologic model, CFIR acknowledges that the effectiveness of interventions implemented within a health system is influenced by the interaction of environmental/policy within the larger health care system in Vietnam, organizational (e.g., organizational readiness), and individual-level factors. Therefore, CFIR organizes constructs into domains that include (a) intervention characteristics, (b) outer setting, (c) inner setting, and (d) individual (patient and provider) characteristics. Wiener et al.’s model addresses “inner setting” or organizational factors positing that implementation strategies (i.e., TC and TCR) enhance implementation effectiveness *through* changes they produce in the implementation climate (i.e., means, opportunity, and expectation) and the extent to which providers perceive that delivering guideline-concordant tobacco use treatment fits their values (i.e., attitudes) and local task requirements (e.g., workload and workflow). ARM 1 (TC) is expected to promote a positive implementation climate by enhancing providers’ perception that tobacco use treatment is a *priority* and by providing the *means* (i.e., new knowledge through training) for adhering to guideline-recommended care. We hypothesize that the addition of a VHW referral system (ARM 2) will result in rates of provider adherence that are superior to ARM 1 through additional changes in perceived *means*, *opportunity*, *and* enhanced *task fit* (i.e., referral system offers providers’ an opportunity to delegate counseling and follow-up).

### Description of intervention conditions

#### ARM 1: provider and staff training and clinical reminder system (TC)

##### Staff training

All clinical and support staff will attend a 2-day training in the use of the 5As and the intervention protocol, including how to make a referral to the VHW (for those in ARM 2). The training is based on the PHS Guideline *Treating Tobacco use and Dependence*, the WHO’s recently released training package for building capacity for tobacco control in primary care and addresses core competences defined by the Association for the Treatment of Tobacco Use and Dependence (http://attudaccred.org/) [5, 30]. Training will be conducted by Dr. Shelley and Dr. Nguyen (MPIs). We will conduct pre- and post-surveys to assess changes in knowledge and to assess satisfaction with the training. A 1-day booster training will be conducted at 3 months after the start of the intervention to address challenges, review screening and counseling techniques, and review documentation procedures.

##### Clinical reminder system and toolkit

The reminder system is designed to remind the intake clinician to assess smoking status, assess readiness, and offer each smoker brief cessation counseling. Given the lack of charts in the CHCs, this component of the intervention was adapted to include posters prompting providers to ask about tobacco use, advice patients to quit, and for sites in the referral arm of the study, to refer patients to the VHW (Additional file 1). Additional materials were developed as part of a larger toolkit to support provider and patient behavior change. These include a table tent provides guidance for providers on how to offer brief counseling and three-patient self-help brochures (dangers of secondhand smoke, health effects of smoking, and creation of a quit plan).

#### ARM 2: TC + referral to VHW

##### Referral to VHW

Based on our pilot research, we created a referral form that is similar to the one we have tested in the USA [16, 17]. For smokers who are interested in quitting and agree to be referred for counseling, the form is completed by the CHC staff. VHWs will then pick up the forms at their regular weekly meetings at the CHCs. Smokers will be contacted within 5 days of their visit to schedule the first of three counseling sessions. The sessions are conducted in person and will last approximately 30 min. The first session will be conducted within 1 week of the patient visit to the CHC (planning session) and the second and third sessions at 2 (within 2 days of quit date) and 4 weeks post-clinic visit. The counseling schedule is based on the PHS Guideline, our pilot data, and evidence that early more intensive contact around the quit date is associated with improved cessation rates [5, 31, 32]. Counseling will focus on motivational barriers for treatment readiness and offer stage-based cessation advice. Motivational interviewing (MI) techniques have been found to be effective in increasing abstinence and quitting attempts [33]. There is also evidence that lay health workers can be trained to offer effective MI [34]. Six VHWs from each intervention clinic will be invited to participate in a 4-day training that will build on the provider training to ensure proficiency in motivational techniques. The MI module was adapted from an existing curriculum and pretested in two focus groups with VHWs. Standardized training, setting performance criteria, and conducting a booster session will help ensure intervention fidelity. In addition, each VHW will have a field manual, which will have all of the information necessary for completion of the intervention components as well as a checklist to complete for each patient interaction. VHWs will receive ongoing supervision by means of bimonthly meetings with trained research staff and will attend a 1-day booster training session 2 months after the intervention begins to minimize drift in counseling skills. Typically, we audiotape counseling sessions to assess fidelity. However, in this setting, we will adapt an approach used by the Vietnam research for previous studies. At the start of each week, VHWs will provide a schedule of home visits to the research assistant (RA). The RA will observe a random selection of the counseling sessions and document delivery of essential components of the VHW protocol using a standardized scoring sheet.

### Evaluation plan

Mixed methods (i.e., qualitative interviews and survey) data will be collected from patients, providers, and VHWs. The evaluation plan is organized according to the following domains: primary outcome, secondary outcome, cost, baseline organization-level moderators, implementation process measures, and implementation fidelity.

### Outcome evaluation (AIM 1)

#### Primary outcome

To assess the primary outcome of provider adherence to tobacco treatment guidelines, we will conduct patient exit interviews (PEI) (surveys conducted immediately after the patient visit) with 50 smokers pre- and 50 post-implementation at each site (1300 in each study period) (Additional file 1) [35–38]. The PEI, completed immediately after the visit, assesses the full spectrum of PHS Guideline recommended care (i.e., 5As). It has well-established validity as evidenced by strong correlation with more costly audiotaped assessment of physician-patient interactions [35]. Prior to and approximately 12 months following each site’s enrollment, consecutive patients will be screened in the waiting area prior to seeing their provider, to determine smoking status and to obtain consent for the exit interview. The PEI will also assess readiness to quit and patient demographics. Patients are eligible to complete a PEI if they are (1) age 18 or over, (2) at the CHC for a routine patient visit, and (2) active smokers (current smoking within the past 7 days).

#### Secondary outcome

All patients who complete a PEI in the pre- and post-intervention period will be followed prospectively to assess 3- and 6-month 7-day point prevalence abstinence, defined as any smoking (even a puff) in the past 7 days [39]. Surveys will be conducted in person and smoking abstinence will be validated using carbon monoxide (CO) monitoring with abstinence defined as a CO < 10 ppm.

#### Cost analysis

Cost measures include implementation costs (e.g., VHW, system changes), patient time costs, and medical costs including model-estimated impacts on downstream costs attributable to tobacco-related illness (e.g., myocardial infarction). Health care and staff salary cost estimates will be derived from Vietnamese health ministry officials. Implementation costs will include health professional time requirements (including training of clinicians and VHWs), major constituents of the VHW intervention, and patient and provider materials. Incremental patient costs will be assessed by surveying additional patient time required by the VHW, as well as any indirect costs that are required (e.g., elder or child care). *Effectiveness inputs* for the simulation will arise from AIM 1 and will include 6-month smoking abstinence and changes in health-related quality of life as measured by the EQ-5D which has been translated into Vietnamese [40]. We will explore variable assumptions regarding the persistence of abstinence (e.g., relapse) in sensitivity analyses. Estimates regarding baseline disease incidence of chronic lung disease, lung cancer, and myocardial infarction, as well as lifetables describing all-cause mortality stratified by sex will be based on Vietnamese data.

#### Baseline organizational characteristics

##### Organizational structure and organizational readiness to change

At baseline, we are collecting data on organizational variables including number of FTE staff, staff characteristics, and clinic volume [28, 41]. We have also adapted a tool developed by Weiner et al., to assess baseline organizational readiness to implement practice guidelines [42]. These measures will be assessed using baseline surveys of medical directors at each CHC.

#### Implementation process evaluation (AIM 2)

We will use a mixed methods approach to assess outer setting (i.e., policies and resources) and inner setting constructs (e.g., climate, resources to support implementation, relative priority) that have been hypothesized to impact implementation outcomes. We have adapted a survey tool developed by Weiner et al., to assess implementation climate, and will use a measure developed by Solberg et al., to assess relative priority for implementing tobacco use treatment into routine practice [27, 43]. Baseline and post-intervention surveys (12 months) administered to clinical staff will assess these constructs. We will also conduct post-implementation semi-structured interviews with a sample of study staff participants in each site (2 VHWs, 3 providers, and 1 Medical Director) to further explore potential barriers and facilitators for tobacco use treatment as defined by CFIR. These include MOH policies, resources (staffing, funding, training), perceptions about the intervention (relative advantage, complexity), and beliefs about their own and their colleagues’ ability to provide effective tobacco use treatment (i.e., self and collective efficacy). The interview guides will also include questions about the process of integrating and customizing the implementation strategy into the CHC workflow as well as acceptability and potential for sustainability.

#### Implementation fidelity

We will use several approaches to evaluate the extent to which each component of the implementation strategies was delivered as intended.

##### Training and clinical reminder systems

RAs will conduct site visits at 2, 6, and 12 months to document (using a checklist) that the reminder and referral systems and other components of the toolkit (e.g., self-help brochures) materials are present and visible in the CHCs. We will record the percentage of staff that attends trainings, as well as any changes in staffing, and we will conduct brief pre- and post-training assessments.

##### Referral system

We will track the number of completed referral forms. We will measure the percentage of patients referred that were reached by the VHW and the number of contacts VHWs made with each patient contacted using VHW logs.

### Analysis plan

#### AIM 1

The primary outcome will be evaluated using mixed-effects regression analysis to estimate the difference between treatment conditions adjusting for the clustering effects across multiple levels (patients, providers, clinics) of the hierarchical data structure [44]. Treatment condition differences after the intervention period will be examined by creating a dummy variable for study arm with the TC condition as a reference category. The fixed-effects coefficient for treatment condition will contrast TCR and TC alone conditions after the intervention period. Covariates such as baseline organizational factors (guideline adherence, FTEs) and urban vs. rural site will be considered for inclusion in the mixed-effects model to reduce the within-group variance, if they are predictive of the primary outcome. A complex error term will be specified with intercepts randomly varying across sites and providers within sites. The secondary outcome is smoking cessation, a binary variable (scored 1 = Yes, 0 = No). We will conduct an intent-to-treat analysis (i.e., those lost to follow-up considered smokers). The basic model again involves a comparison of the two implementation strategies (TC vs. TCR). The binary outcome will be modeled by a generalized linear mixed-effects model similar to what was described above for the primary outcome [44]. A binomial family distribution and logit link will be used. Exponentiating the fixed-effects coefficient for treatment condition will generate an estimate of the odds ratio (i.e., the effect of treatment condition on the odds of patient smoking cessation).

#### Power

For the primary outcome, optimal design [45] was used to estimate power to detect an effect size of one standard deviation. We assume 10 patients per clinician, 5 clinicians per site, and, conservatively, 33 % variance (i.e., the intra class correlation or ICC) at each level (site, clinician, patient). With 26 sites and assuming a two-sided type-1 error of 0.05, power is approximately 0.95 to detect this effect size. Optimal design was also used to estimate power for the secondary outcome, where an odds ratio of 2.3 is expected (13 % vs. 26 % abstinence for TC and TCR conditions, respectively) [5]. We assume a plausible interval for the smoking cessation rate among patients in the TC alone condition ranging from 0.01 to 0.25, and we assume conservatively that 50 % of the variability in cessation rates is between clinicians. With 26 sites, we have 80 % power to detect the estimated effect size.

Cost-effectiveness analysis (CEA): We will estimate the incremental cost-effectiveness (ICER) of ARM 2 compared to ARM 1 which is defined as the incremental change in costs divided by the incremental change in effectiveness (e.g., QALYs with TCR minus QALYs with TC alone). “QALY” refers to quality-adjusted life expectancy and is a quantitative measure that simultaneously takes into account both quality and quantity of life. We will use measures of effect based on analyses from this study combined with mathematical modeling to inform the derivation of the cost and QALY estimates over longer time horizons than those over which data will be collected (5-year, 10-year, 20-year, and lifetime horizons). Downstream impact of TCR on future costs and benefits (e.g., the downstream health impact of averting chronic lung disease, as well as the downstream costs averted by preventing those diseases) will be assessed via mathematical modeling using a Markov (“state-transition”) computer simulation that will enable attribution of the health benefit that would be caused by specified reductions in smoking [46–49]. We will perform the analysis from both a societal perspective and from a payer perspective [50].

#### AIM 2

For quantitative analysis, mixed-effects models for AIM 2 will be expanded to include additional main and interaction effects. A mixed-effects model also will be estimated for implementation climate and fit with providers’ values and tasks, to determine whether treatment conditions were different on the hypothesized mediators. These hypothesized mediators also will be added to the models for AIM 2, to determine whether climate and fit with providers’ values and tasks have unique effects on outcomes, after controlling for treatment condition and covariates. Baron and Kenny’s [51] causal-steps test can be applied to explore if the impact of implementation strategies on guideline adherence operates through implementation climate and/or innovation fit. Multilevel structural equation modeling also will be employed to estimate indirect effects of treatment condition on guideline adherence [52–55]. Effect modification by baseline site (i.e., organizational readiness) characteristics will be explored by adding interaction effects between potential modifiers and treatment condition. Qualitative data obtained from the audio recordings of the semi-structured interviews and focus groups will be transcribed verbatim in Vietnamese by ISMS and then translated into English. Investigators’ fluency in English and Vietnamese will address concerns about ensuring “conceptual equivalence” [56, 57]. A coding manual will be developed and finalized in an iterative process. All transcripts will then be independently coded by two members of the research team to establish inter-rater reliability.

### Ethical review

The New York University School of Medicine and ISMS Institutional Review Boards have approved the study.

### Trial status

Funded in September 2013, in year 1, we finalized the training curriculum and provider/VHW training plan, finalized toolkit materials including the self-help brochures and clinical reminder system (poster and table tents), and finalized the survey tools and evaluation plan. We have recruited all 26 CHCs and completed baseline assessment for Wave 1 in August 2014. The baseline assessment included collecting 398 PEIs, completing surveys with 50 clinical providers and 147 VHW, and semi-structured interviews with 40 providers and VHWs (5 at each CHC). The Wave 1 intervention period started with the provider and VHW trainings in September 2014. Consistent with the protocol, 12-month post-intervention PEIs will be completed in October 2015. Wave 2 (10 CHCs) was launched in July 2015 and to date we have completed 401 baseline PEIs.

## Discussion

Tobacco use continues to be the leading global cause of preventable deaths. Of the world’s 1.25 billion adult smokers, 10 % reside within SEACs [58]. Therefore, it is critical to develop strategies for increasing access to evidence-based tobacco use treatment services that could lead to significant reductions in tobacco-related morbidity and mortality. The WHO, through the FCTC, has started to take action to reduce the burden of tobacco-related disease in LMICs [8]. Treatment of tobacco use is mandated in Article 14 of the WHO FCTC as a key component of comprehensive tobacco control strategy. Tobacco dependence treatment is also recommended by the WHO as part of a comprehensive package of essential services for prevention and control of non-communicable diseases (NCDs) in primary care in accordance to the revised draft of the WHO Global Action Plan for the Prevention and Control of NCDs (2013–2020). Yet, there are tremendous gaps in knowledge regarding how to implement guidelines for tobacco use treatment in public health systems in LMICs. We are not aware of any research that has attempted to systematically study strategies for implementing tobacco use treatment guidelines as a routine part of care primary care in LMICs. The current study has the potential to provide relevant information to guide large-scale adoption of strategies for implementing and disseminating tobacco use treatment guidelines throughout the public health system in Vietnam and to serve as a model for similar action in other LMICs and the U.S.

There are some potential limitations. First, we have defined the core elements of the implementation strategies; however, we acknowledge that adaptations to the unique practice context will be necessary. We will use fidelity checks to ensure that the core elements are implemented and will document adaptations to enhance external validity. Second differences between high-income and low-income health care systems, including the high smoking rates among male physicians, may pose challenges for implementation. However, our pilot research, which is consistent with national surveys, found that smoking rates among women (nurses and doctors) is less than 5 % [1]. Over 70 % of providers working in CHCs and 90 % of VHWs are female, somewhat mitigating this potential problem. Finally, cessation medication is not available in Vietnam; however, there is good evidence that brief counseling alone from a physician or other health care professional can increase abstinence rates by 30 % and more intensive counseling, even without medication, can result in quit rates of >20 % at 6 months compared to less than 5 % without treatment [8]. In order to test the impact of an intervention that would have the most potential for sustainability, we are not providing cessation pharmacotherapy.

Despite limitations, the findings have potential for high impact by identifying best practices for implementing tobacco use treatment in public health care delivery systems in Vietnam and other LMICs and providing key stakeholders with the data they need to make decisions regarding dissemination of effective tobacco dependence treatment guidelines.

## Additional file

Additional file 1:
**Patient exit interview (PEI).** (DOCX 106 kb)

## Abbreviations

CFIR: consolidated framework for implementation research
CEA: cost-effectiveness analysis
CHCs: community health centers
ICER: incremental cost-effectiveness
LMICs: Low middle-income countries
MPI: multiple principal investigator
NCDs: non-communicable diseases
PEI: patient exit interview
PHS: public health service
QALY: quality-adjusted life years
SEAC: Southeast Asian countries
TC: training and clinical reminder system
TCR: training, clinical reminder system and referral
VHW: village health worker
WHO: World Health Organization
