# Supplementary material for: Implementing tobacco use treatment guidelines in community health centers in Vietnam
Source: Implement Sci. 2015 Oct 9;10:142. doi: 10.1186/s13012-015-0328-8 (PMC4600252; doi:10.1186/s13012-015-0328-8)
Supplement: Additional file 1: — Patient exit interview (PEI). (DOCX 106 kb) [file 13012_2015_328_MOESM1_ESM.docx]

**Patient Exit Interview (PEI)**

Starting time:………hour……..minute

|  | | **A – Identification** | | | | | |  |
| --- | --- | --- | --- | --- | --- | --- | --- | --- |
| **No** | | **Question** | | **Code** | | | | **Skip** |
| 1. A0 | | Date of interview | |  | | | |  |
| 1. 1 | | District | | ……………………………………………………… | | | |  |
| 1. 3. | | Commune | | ……………………………………………………… | | | |  |
|  | | Village | | ……………………………………………………… | | | |  |
|  | | Name and code of respondent | | ……………………………………………………… | | | |  |
| 1. 3 | | Name and code of interviewer | | ……………………………………………………… | | | |  |
| 1. 4 | | Name and code of supervisor | | ……………………………………………………… | | | |  |
| **B – Demographic Characteristics** | | | | | | | | |
|  | | How old are you? | |  | | | |  |
|  | | Gender (observe and write down the sex of interviewee) | | Male 1  Female …….2 | | | |  |
|  | | What is the highest level of education you have achieved? | | Not complete primary school 1  Primary school 2  Secondary school 3  High school 4  Vocational training/ College 5  University 6  Graduate 7 | | | |  |
|  | | How long have you lived in commune? | | Year Month | | | |  |
|  | | How would you describe your ethnicity? | | Kinh 1  Other 2 | | | |  |
|  | | What is your marital status? | | Single/Never married ………………… 1  Married 2  Divorced 3  Separated 4  Widowed 5  Other 6 | | | |  |
|  | | What is your current **main** job? | | Farmer (farming, livestock, cultivation)/Fishermen .1  Officers and employees (state, locality) 2  Working for non-state agencies (including workers) 3  Small businessmen/Craft/Trading/Services (tailor, hairdresser, mason)/Freelance 4  Homemaker/Non-worker/Student/Pupil 5  Other (specify: ____________________) 8 | | | |  |
|  | | What was your total household income during the past 12 months?  (total income of every family member calculated in VND) | | <2,000,000 1  2,000,000 - <10,000,000 2  10,000,000 - <50,000,000 3  50,000,000 - <100,000,000 4  100,000,000 - 300,000,000 5  More than 300,000,000 6  Do not know 8 | | | |  |
| **C – Main content** | | | | | | | | |
|  | | Reason for visit  (Check All That Apply) | | Examination for a new problem…………………. 1  Follow-up…………………………...……………. 2  Getting referral to a higher level of care …...……. 3  Routine checkup ………..………………………… 4  Other (specify)………………………….……..….. 6 | | | |  |
|  | | **Which main** health care provider did you see today?) | | Doctor ………………………………………………1  Physician assistant…………………………………..2  Nurse………………………...………………………3  Midwife …………………………..............................4  Pharmacist…………………………………………...5  Other (specify)…………………………………..….6 | | | |  |
| 3a. | | Do you smoke cigarettes some days, every day or not at all? | | Some days 1  Every day 2  Not at all 3 | | | | If 3 skip to 4.a |
| 3.b | | On the days that you smoke cigarettes how many do you smoker per day? | | # cigarettes | | | |  |
| 3.c | | How soon after you awake do you smoke your first cigarette? | | _5 min or less 1  6–30 min 2  31–60 min 3  61 min or more 4 | | | |  |
| 4.a | | Do you smoke a waterpipe somedays every day or not at all? | | Some days 1  Every day 2  Not at all 3    ***Note for interviewer: If not using cigarettes or waterpipe not eligible for interview*** | | | | **If 3 go to C5** |
| 4.b | | On the days that you smoke, how many times a day do you smoke a waterpipe? | |  | | | |  |
| 4.c | | How soon after you awake do you smoke your first time of waterpipe? | | _____ minutes ____ hours | | | |  |
|  | | **The following questions ask about your visit today** | | | | | |  |
| 5. | | Did your health care provider ask you if you smoked cigarettes or water pipes? | | Yes….……………………………………………….1  No………………………………………...…………2 | | | |  |
| 6. | | Did your health care provider advise you to quit smoking cigarettes or waterpipe? | | Yes….……………………………………………….1  No………………………………………...…………2 | | | |  |
| 7. | | Did your health care provider **ask you if you were ready to quit** smoking cigarettes or waterpipe? | |  | | | |  |
| 8a. | | Did you tell your health provider that you were ready to quit smoking or reduce the use of cigarettes/waterpipe? | | Yes….……………………………………………….1  No………………………………………...…………2 | | | |  |
| 8b. | | Did your health care provider **provide counseling** to help you to quit smoking cigarettes or waterpipe? | | Yes….……………………………………………….1  No………………………………………...…………2 | | | |  |
| 9 | | Did your health provider give you any written information about how to quit smoking cigaerttes or waterpipe? | | Yes….……………………………………………….1  No………………………………………...…………2 | | | |  |
| 10 | | Did your health provider talk to you about the use of medication to help you quit smoking cigarettes or or waterpipe? | | Yes….……………………………………………….1  No………………………………………...…………2 | | | |  |
| 11 | | Did your health provider ask you if you wanted him/her to refer you to a Village Health worker for additional help to quit smoking cigarettes or waterpipe? | | Yes….……………………………………………….1  No………………………………………...…………2 | | | | **2⮷C13** |
| 12 | | Did you agree to be referred? | | Yes….……………………………………………….1  No………………………………………...…………2 | | | |  |
| 13 | | Did your health provider give you a referral or the phone number to another smoking cessation program to help you quit smoking cigarettes or waterpipe | | Yes….……………………………………………….1  No………………………………………...…………2 | | | |  |
| 14 | | During the past year have you tried to stop smoking cigarettes or waterpipe? | | Yes, Cigarettes 1  Yes, Waterpipe 2  Yes, Both cigarettes and waterpipe 3  No ……………………………………………..4 | | | |  |
|  | | | **Strongly Disagree** | | **Disagree** | **Agree** | **Strongly Agree** |  |
| 15. | How much do you agree or disagree with this statement? There is no right or wrong answer. Choose only one answer per question. **(Use Show card, read the statement, and ask for interviewee to chose on the point scale)**  People who are important to you believe that you should not smoke | | 1 | | 2 | 3 | 4 |  |
| **D- INFORMATION FOR FOLLOW UP SURVEYS** | | | | | | | | |
| 16. | | First and last name: | | …………………………………………….. | | | |  |
| 17. | | Address: | | a. Household Number and street: …………………..  b. Village ……………………………..……………..  c. Commune: ……………………………………….. | | | |  |
| 18. | | Phone numbers (please provide three different contact numbers) | | 1a. Cell (1): ………………………………………….  1b. Cell (2): ………………………………………….  2. Home: ……………..………………………………    3. Work: …………………..………………………… | | | |  |
| 19. | | E-mail address: | | …………………………………………………… | | | |  |

Ending time: ………hour………minute

**Thank you for taking the time to complete this survey!**
